# Red cell distribution width is correlated with extensive coronary artery disease in patients with diabetes mellitus

**DOI:** 10.5830/CVJA-2017-015

**Published:** 2017

**Authors:** Atac Celik, Metin Karayakali, Fatih Altunkas, Kayihan Karaman, Arif Arisoy, Koksal Ceyhan, Hasan Kadi, Fatih Koc

**Affiliations:** Department of Cardiology, Faculty of Medicine, Gaziosmanpasa University, Tokat, Turkey; Department of Cardiology, Faculty of Medicine, Gaziosmanpasa University, Tokat, Turkey; Department of Cardiology, Faculty of Medicine, Gaziosmanpasa University, Tokat, Turkey; Department of Cardiology, Faculty of Medicine, Gaziosmanpasa University, Tokat, Turkey; Department of Cardiology, Faculty of Medicine, Gaziosmanpasa University, Tokat, Turkey; Department of Cardiology, Faculty of Medicine, Gaziosmanpasa University, Tokat, Turkey; Department of Cardiology, Faculty of Medicine, Balikesir University, Balikesir, Turkey; Department of Cardiology

**Keywords:** red cell distribution width, coronary artery disease,, diabetes mellitus, Gensini score

## Abstract

**Introduction:**

Previous studies have predicted an independent relationship between red cell distribution width (RDW) and the risk of death and cardiovascular events in patients with coronary artery disease (CAD). The aim of this study was to investigate the relationship between RDW and extensiveness of CAD in patients with diabetes mellitus (DM).

**Methods:**

Two hundred and thirty-three diabetic patients who underwent coronary angiographies at our centre in 2010 were included in the study. All of the angiograms were re-evaluated and Gensini scores were calculated. Triple-vessel disease wasdiagnosed in the presence of stenosis > 50% in all three coronary artery systems.

**Result:**

RDW was significantly higher in diabetic CAD patients (p < 0.001). Patients with CAD who had a RDW value above the cut-off point also had higher Gensini scores, higher percentages of obstructive CAD and triple-vessel disease (p ≤ 0.001 for all). According to the cut-off values calculated using ROC analysis, RDW > 13.25% had a high diagnostic accuracy for predicting CAD. RDW was also positively correlated with Gensini score, obstructive CAD and triple-vessel disease (r < 0.468 and p < 0.001 for all).

**Conclusion:**

RDW values were found to be increased in the diabetic CAD population. Higher RDW values were related to more extensive and complex coronary lesions in patients with DM.

## Introduction

Red cell distribution width (RDW) is widely accepted as a measure of anisocytosis and is routinely reported during automated complete blood counts.^1^ It is commonly used to narrow the differential diagnosis of anaemia.^2^ Many studies have reported that higher RDW values are associated with a worse prognosis in coronary artery disease, heart failure, peripheral artery disease, and even in the unselected population.^3^-^6^

Diabetes mellitus (DM) is one of the major risk factors for atherosclerosis.^7^ Coronary artery disease (CAD) is more common among patients with DM.^8^ CAD is the main cause of death in DM, and DM is associated with a two- to four-fold increased mortality risk from heart disease.^9^ Moreover, it has a worse prognosis and is usually more advanced at the time of diagnosis.^10^

Previous studies have shown an association between RDW value and the severity of CAD, but there were no data on the diabetic population.^11^-^13^ The aim of this study was to investigate the relationship between RDW and the extensiveness of CAD in patients with DM.

## Methods

The study group was formed retrospectively from our catheterisation laboratory registries. Two hundred and thirtythree diabetic patients who underwent coronary angiography at our centre in 2010 were included in the study. The diagnosis ofDM was based on a previous history of diabetes treated with or without drug therapies.

Patients with acute or chronic inflammatory disease, severe liver or renal insufficiency, morbid obesity, malignancy, valvular heart disease, heart failure, prior coronary intervention, or who had experienced acute coronary syndrome within 30 days prior to coronary angiography were excluded from the study. In addition, subjects were also excluded if they had a history of anaemia and blood transfusion.

Patient age, gender, past history of disease, smoking habits and current medications were carefully ascertained. Hypertension was defined as blood pressure ≥ 140/90 mmHg or if the subject was taking antihypertensive medications. Dyslipidaemia was defined as low-density lipoprotein cholesterol ≥ 100 mg/dl (≥ 2.59 mmol/l) or if they were taking a hypolipidaemic drug. Anaemia was defined as haemoglobin concentration < 13 mg/dl in men and < 12 mg/dl in women. Body mass index (BMI) was calculated as weight/height2 (kg/m2).

This investigation was a single-centre study. Informed consent was obtained from all participants, and the study protocol was approved by the ethics committee at our institution. The study was in accordance with the Declaration of Helsinki.

Blood samples were drawn from each patient after overnight fasting, during admission for routine chemistry. Haemoglobin, white blood cell count, mean platelet volume (MPV) and RDW values were measured with a Pentra DX 120 analyser (ABX, Montpellier, France). Neutrophil/lymphocyte (N/L) ratio was calculated by dividing the total neutrophil count by the lymphocyte count.

High-sensitivity C-reactive protein (hs-CRP) analyses were done using the immunonephelometry method (Dade Behring, Inc, BN Prospect, Marburg, Germany). Serum levels of creatinine, fasting blood glucose, triglycerides, total cholesterol, and low- and high-density lipoprotein cholesterol were measured using conventional methods.

A conventional angiography device (Artis zee; Siemens, Erlangen, Germany) was used for coronary angiography. Angiograms were evaluated qualitatively by two different experts, and mean values were used to assess the rate of stenosis. Patients with atherosclerotic lesions in any of the coronary arteries were diagnosed as having CAD. Obstructive CAD was defined as stenosis of ≥ 50% of the diameter of a major epicardial or branch vessel > 2.0 mm in diameter.

Gensini scores were calculated for each patient as previously defined.^14^ Triple-vessel disease was defined as stenosis of ≥ 50% in each of the major vessels or their 14major branches. Patients were evaluated and treated according to the current guidelines.

## Statistical analysis

Statistical analysis was performed using commercial software (IBM SPSS Statistics 22, SPSS Inc, Chicago, IL, USA). After performing the Kolmogorov–Smirnov normality test, two independent-sample t-tests were used to compare the normally distributed independent variables, and the Mann–Whitney U-test was used to compare the non-normally distributed independent variables between the two groups. For normally distributed variables, mean and standard deviation (SD) are listed, otherwise, median values are given. To analyse the categorical data, a chi-squared test was used. Categorical data are expressed as numbers and percentages.

A receiver operating characteristic (ROC) curve was constructed for RDW to test the effectiveness of various cut-off points in predicting CAD. The area under the ROC curve was calculated; the sensitivity and specificity for the RDW of the most appropriate cut-off point were calculated for predicting CAD. Correlations were determined using the Spearman test. A p-value < 0.05 was considered statistically significant.

## Results

The study group was divided into two, according to angiographic results (CAD negative and CAD positive). There were no significant differences between the two groups with regard to age, gender, hypertension, hyperlipidaemia, smoking, BMI, systolic and diastolic blood pressure, and medications, including aspirin, renin–angiotensin system (RAS) blockers and statins (Table 1).

**Table 1:** Baseline characteristics and laboratory findings of the study groups

*Variables*	*CAD–(n = 109)*	*CAD+(n = 124)*	*p-value*
Age (years)	58.6 ± 8.0	57.7 ± 9.0	0.387
Gender (male)	61 (56)	68 (55)	0.895
Hypertension	93 (85)	104 (84)	0.856
Dyslipidaemia	61 (56)	77 (62)	0.353
Smoking	14 (13)	24 (20)	0.215
Aspirin	72 (66)	93 (75)	0.150
Clopidogrel	0 (0)	23 (19)	< 0.001
RAS blockers	70 (64)	93 (75)	0.086
β-blockers	34 (31)	66 (53)	0.001
Calcium channel blockers	20 (18)	23 (19)	1.000
Statins	30 (28)	43 (38)	0.260
Body mass index (kg/m^2^)	28.7 ± 5.0	28.3 ± 4.5	0.536
Systolic blood pressure (mmHg)	130 ± 13	132 ± 14	0.144
Diastolic blood pressure (mmHg)	78 ± 9	79 ± 8	0.627
Glucose (mg/dl)	166 ± 75	174 ± 78	0.416
[mmol/l]	[9.21 ± 4.16]	[9.66 ± 4.33]	
Creatinine (mg/dl)	0.73 ± 0.18	0.71 ± 0.28	0.630
[μmol/l]	[64.53 ± 15.91]	[62.76 ± 24.75]	
Uric acid (mg/dl)	4.5 ±1.4	4.9 ± 1.7	0.081
hs-CRP (mg/l)	5.12 ± 2.93	6.07 ± 4.83	0.348
Total cholesterol (mg/dl)	197 ± 40	199 ± 49	0.726
[mmol/l]	[5.10 ± 1.04]	[5.15 ± 1.27]	
Triglycerides (mg/dl)	187 ± 86	191 ± 138	0.786
[mmol/l]	[2.11 ± 0.97]	[2.16 ± 1.56]	
LDL cholesterol (mg/dl)	120 ± 36	122 ± 44	0.688
[mmol/l]	[3.11 ± 0.93]	[3.16 ± 1.14]	
HDL cholesterol (mg/dl)	46 ± 11	45 ± 13	0.283
[mmol/l]	[1.19 ± 0.28]	[1.17 ± 0.34]	
WBC (10^3^ cells/μl)	7.0 ± 1.9	7.2 ± 2.0	0.407
Haemoglobin (g/dl)	13.1 ± 1.1	13.1 ± 1.6	0.757
RDW (%)	12.5 ± 1.5	13.8 ± 1.7	< 0.001
MPV (fl)	8.43 ± 1.10	8.59 ± 1.02	0.265
Neutrophil/lymphocyte ratio (%)	2.26 ± 1.37	2.52 ± 1.94	0.457

CAD: coronary artery disease, CAD–: patients with normal coronary arteries, CAD+: patients with coronary artery disease, RAS: renin–angiotensin system, hs-CRP: high-sensitivity C-reactive protein, LDL: low-density lipoprotein, HDL: high-density lipoprotein, WBC: white blood cells, RDW: red cell distribution width, MPV: mean platelet volume. Data are shown as n (%) or mean ± SD

Clopidogrel and calcium channel blocker use was higher in the CAD-positive group (p < 0.001 and p = 0.001, respectively) (Table 1). There were no differences between the two groups in serum levels of glucose, creatinine, uric acid, hs-CRP, lipid profile, WBC, haemoglobin, MPV and N/L ratio (Table 1). RDW was significantly higher in the CAD-positive group (12.5 ± 1.5 vs 13.8 ± 1.7%, p < 0.001) (Table 1).

The most appropriate cut-off point calculated for predicting CAD was 13.25%. The patients who had a RDW ≤ 13.25% were included in the low RDW group. The rest formed the high RDW group.

There were no significant differences between the low and high RDW groups with regard to age, gender, hypertension, hyperlipidaemia, smoking, BMI, systolic and diastolic blood pressure and medications (Table 2). There were also no differences between the low and high RDW groups with regard to serum levels of glucose, uric acid, lipid profile, WBC and haemoglobin (Table 2).

**Table 2:** Baseline characteristics and laboratory findings of low and high RDW groups

*Variables*	*Low RDW (≤ 13.25) (n = 46)*	*High RDW (> 13.25) (n = 78)*	*p-value*
	56.7 ± 8.0	58.2 ± 9.5	0.381
Gender (male)	27 (59)	41 (53)	0.318
Hypertension	38 (83)	66 (85)	0.478
Dyslipidaemia	29 (63)	48 (61)	0.511
Smoking	5 (11)	19 (24)	0.052
Aspirin	33 (72)	60 (77)	0.331
Clopidogrel	11 (24)	12 (15)	0.173
RAS blockers	32 (70)	61 (78)	0.195
β-blockers	28 (61)	38 (49)	0.130
Calcium channel blockers	9 (20)	14 (18)	0.501
Statins	13 (28)	30 (39)	0.169
Body mass index (kg/m^2^)	28.8 ± 4.5	28.0 ± 4.5	0.363
Systolic blood pressure (mmHg)	131 ± 13	133 ± 15	0.328
Diastolic blood pressure (mmHg)	78 ± 8	79 ± 8	0.196
Glucose (mg/dl)	163 ± 77	181 ± 79	0.207
[mmol/l]	[9.05 ± 4.27]	[10.05 ± 4.38]	
Creatinine (mg/dl)	0.63 ± 0.17	0.76 ± 0.31	0.008
[μmol/l]	[55.69 ± 15.03]	[67.18 ± 27.40]	
Uric acid (mg/dl)	4.6 ± 1.5	5.1 ± 1.7	0.213
hs-CRP (mg/l)	4.11 ± 1.88	7.12 ± 5.58	0.043
Total cholesterol (mg/dl)	195 ± 44	202 ± 52	0.481
[mmol/l]	[5.05 ± 1.14]	[5.23 ± 1.09]	
Triglycerides (mg/dl)	197 ± 173	188 ± 114	0.736
[mmol/l]	[2.23 ± 1.95]	[2.12 ± 1.29]	
LDL cholesterol (mg/dl)	114 ± 33	127 ± 48	0.088
[mmol/l]	[2.95 ± 0.85]	[3.29 ± 1.24]	
HDL cholesterol (mg/dl)	46 ± 15	44 ± 12	0.461
[mmol/l]	[1.19 ± 0.39]	[1.14 ± 0.31]	
WBC (10^3^ cells/μl)	7.1 ± 1.9	7.3 ± 2.2	0.516
Haemoglobin (g/dl)	13.3 ± 1.5	13.0 ± 1.6	0.454
RDW (%)	12.9 ± 0.7	14.3 ± 1.4	0.001
MPV (fl)	8.35 ± 1.13	8.72 ± 0.93	0.049
Neutrophil/lymphocyte ratio (%)	1.92 ± 0.07	2.89 ± 2.33	0.009

RDW: red cell distribution width, RAS: renin–angiotensin system, hs-CRP: high-sensitivity C-reactive protein, LDL: low-density lipoprotein, HDL: highdensity lipoprotein, WBC: white blood cells, MPV: mean platelet volume. Data are shown as n (%) or mean ± SD

Serum levels of creatinine, hs-CRP, MPV and N/L ratio were significantly higher in the high RDW group (p < 0.005 for all) (Table 2). RDW was positively correlated with hs-CRP, MPV and N/L ratio (r = 0.248, r = 0.240 and r = 0.281, respectively and p = 0.033 for hs-CRP, p < 0.001 for MPV and N/L ratio).

**Fig. 1.**
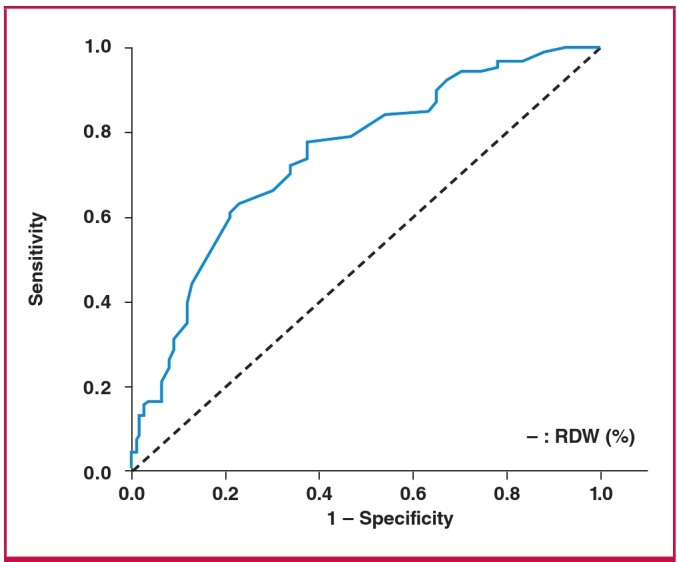
Receiver operating characteristic curve showing the relationship between sensitivity and false positivity at various cut-off points for red cell distribution width to predict coronary artery disease.

Patients with CAD who had a RDW value above the cut-off point also had higher Gensini scores, higher percentages of obstructive CAD and triple-vessel disease (p ≤ 0.001 for all) (Table 3). According to the cut-off values calculated using ROC curve analysis, RDW > 13.25% had a high diagnostic accuracy for predicting CAD (area under the ROC curve = 0.742, p < 0.001) (Table 4, Fig. 1). RDW was positively correlated with Gensini score, obstructive CAD and triple-vessel disease (r = 0.468, r = 0.409 and r = 0.332, respectively and p < 0.001 for all).

**Table 3:** Severity of coronary artery disease between low and high RDW groups

*Variables*	*Low RDW (≤ 13.25) (n = 46)*	*High RDW (> 13.25) (n = 78)*	*p-value*
Gensini score			
Total	11 [4–31]	43 [16–73]	< 0.001
LAD	5 [3–12]	18 [5-30]	0.001
Cx	3 [1–5]	7 [3–19]	< 0.001
RCA	2 [1–3]	7 [2–18]	< 0.001
Obstructive CAD	23 (50)	63 (81)	0.001
Triple-vessel disease	2 (4)	26 (33)	< 0.001

RDW: red cell distribution width, LAD: left anterior descending coronary artery, Cx: circumflex coronary artery, RCA: right coronary artery, CAD: coronary artery disease. Data are shown as n (%) or median [interquartile range].

**Table 4:** Diagnostic accuracy of red cell distribution width for coronary artery disease

*Variable*	*Cut-off value*	*AUC*	*95% CI of AUC*	*Sensitivity*	*Specificity*	*p-value^a^*
RDW (%)	> 13.25	0.742	0.679–0.806	0.629	0.771	< 0.001

AUC: area under the receiver operating characteristic curve, CI: confidence interval, RDW: red cell distribution width. aSignificance level of AUC.

## Discussion

This study showed an association between RDW and CAD in diabetic patients. RDW values were found to be higher in the diabetic CAD population and higher RDW values were related to more extensive and complex coronary lesions.

RDW is a marker of the variation in size of red blood cells circulating in the body, which reflects the value of anisocytosis.^1^ It is routinely reported during automated complete blood counts. An elevation in RDW values may be seen in patients with ineffective erythropoiesis (iron, vitamin B_12_ or folic acid deficiency and various haemoglobinopathies), recent blood transfusions and haemolysis.^15^ In daily practice it is commonly used to narrow the differential diagnosis of anaemia.^2^

The growing attention given to the relationship between RDW and cardiovascular events was first spurred on by the report from Felker et al., which concluded that there was a strong and independent association between RDW and the risk of adverse outcomes in heart failure patients.^16^ Subsequently, Tonelli et al. predicted an independent relationship between RDW and the risk of cardiovascular death in patients with CAD.^3^,^16^ Following the direction of these studies, researchers reported that higher RDW values were also associated with a worse prognosis in peripheral artery disease and even in the unselected population.^5^,^6^

Several explanations could be postulated in order to explain the underlying mechanisms that may contribute to a worse prognosis among patients with cardiovascular disease. However the reason for the poor prognosis remains unclear.

It has not been determined yet whether RDW is a marker of the severity of various disorders or if there is direct link between anisocytosis and poor prognosis in patients with CAD. Factors airing bone marrow haematopoiesis are probably identical to those that worsen the prognosis in CAD. These factors are anaemia, iron deficiency, lipid disorders, chronic inflammation, neurohumoral activation, glycaemic disturbance, vitamin D3 deficiency, oxidative stress and renal failure.^17^,^13^ Additionally, red cell deformability diminution may result in impaired flow through the microcirculation.^17^

Previous studies have shown an association between RDW and the severity of CAD.^11^-^13^ Akin et al. investigated the association of RDW with the severity of CAD in acute myocardial infarction and showed that higher RDW values were correlated with higher Syntax scores, which means more complex coronary lesions. They found that after multiple logistic regression analysis, RDW remained a significant predictor for the severity of CAD.^11^ Isik et al. evaluated this relationship in patients with stable angina pectoris and found an independent association between RDW and the complexity of CAD, which was determined with Syntax scores.^12^

A large Chinese cohort study with 677 subjects showed significantly elevated RDW values in CAD patients and a positive correlation between RDW and the Gensini score.^13^ They also found that a RDW value of 12.85% was an effective cut-off point for predicting CAD, with a sensitivity of 50% and a specificity of 65%. Recently, Sahin et al. concluded that RDW values were independently associated with a high Syntax score but were not associated with long-term mortality in patients with non-ST-elevation myocardial infarction.^19^

In agreement with the current literature, we found that elevation in RDW values was associated with both the presence and complexity of CAD. Furthermore, we found that an RDW value of 13.25% was an effective cut-off point in order to determine the presence of CAD. Moreover, our study is the first to show an association between RDW and CAD severity in a diabetic population.

Chronic inflammation and neurohumoral activation are thought to be the key factors for both a worse cardiovascular prognosis and more complex coronary lesions.^17^,^18^ In our study, hs-CRP levels were similar in the two CAD groups, but there was a positive correlation between RDW and hs-CRP. Unfortunately, we did not measure brain natriuretic peptides, which are markers of the neurohumoral pathway. Some researchers demonstrated that elevated mean platelet volume (MPV) was associated with acute coronary syndromes, thrombosis and inflammation.^20^,^21^ We also found a positive relationship between RDW and MPV.

It is well known that there is a link between glycaemic disturbance and high RDW values. Two different studies showed a relationship between glycosylated haemoglobin and RDW in an unselected elderly population and in healthy adults.^22^,^23^ Garg et al. demonstrated that glycosylated haemoglobin was an independent predictor of CAD severity in a non-diabetic population.^24^ Our findings support the results of previous studies.

This study has some limitations. First, we did not measure some factors that might have influenced RDW levels, such as vitamin B_12_, folate and iron levels. Second, cardiovascular events were not analysed due to the cross-sectional nature of the study. Third, the relationship between RDW, glycaemic disturbance and the severity of CAD could have been better understood if we had analysed glycosylated haemoglobin levels. Lastly, the diagnosis of DM was based on a previous history instead of biochemical results.

## Conclusion

RDW values were significantly higher in diabetic than non-diabetic patients with CAD. Higher RDW values were related to more extensive and complex coronary lesions, suggesting that RDW may be a marker for predicting CAD severity in patients with DM.
